# Real-world data of six patients with atypical hemolytic uremic syndrome switched to ravulizumab

**DOI:** 10.1007/s00467-021-05203-8

**Published:** 2021-07-17

**Authors:** Rasmus Ehren, Sandra Habbig

**Affiliations:** Pediatric Nephrology, Children’s and Adolescents’ Hospital, Department of Pediatrics, Faculty of Medicine and University Hospital Cologne, University of Cologne, Kerpener Str 62, 50937 Cologne, Germany

To the editors,

Ravulizumab, a long-acting C5-inhibitor, has been shown to be efficacious and safe in clinical studies in pediatric patients with preceding eculizumab treatment [1] and in therapy-naïve pediatric and adult patients [2, 3] with atypical hemolytic uremic syndrome (aHUS).

We present here the first real-world data of six pediatric patients with genetically proven aHUS switched to ravulizumab after a median time of 69 (range 6–123) months of eculizumab treatment. Four patients were diagnosed with a complement factor H (CFH) mutation, one patient with a complement factor 3 (C3) mutation, and one with DEAP-HUS (compare Supplementary Table 1 for detailed patient characteristics). Hematological and renal parameters remained stable as shown exemplarily for kidney function and lactate dehydrogenase (LDH) (Supplementary Figure 1). Comprehensive complement surveillance showed stable AP50 suppression and suppressed sC5b-9 levels 3 months after therapy switch as compared to before (Supplementary Figure 1). None of the patients reported any side effects of ravulizumab treatment in the current investigation interval of a median of 220 (range 90–274) days. Importantly, all patients reported a subjective benefit in quality of life due to the extended dosing interval.

Our data support the conclusion of Tanaka et al. [1] and add six definitely diagnosed patients with aHUS to the existing evidence that ravulizumab is effective and safe in pediatric patients. Thus, our data are a significant contribution to the growing body of evidence. In addition, we were able to show that the switch to ravulizumab is feasible in a real-life setting.

## Supplementary Information

ESM 1 (PDF 668 kb)

ESM 2 (PDF 561 kb)

## Data Availability

Not applicable. Not applicable.
