# Supplementary material for: Real-world data of six patients with atypical hemolytic uremic syndrome switched to ravulizumab
Source: Pediatr Nephrol. 2021 Jul 17;36(10):3281–2. doi: 10.1007/s00467-021-05203-8 (PMC8445866; doi:10.1007/s00467-021-05203-8)
Supplement: Supplementary file 1 — (PDF 668 kb) [file 467_2021_5203_MOESM1_ESM.pdf]

Letter to the Editors

Real-world data of six patients with atypical hemolytic uremic syndrome switched to ravulizumab

Rasmus Ehren[1], Sandra Habbig[1]

[1] University of Cologne, Faculty of Medicine and University Hospital Cologne, Department of Pediatrics, Cologne, Germany

Corresponding author

Dr. Rasmus Ehren

Pediatric Nephrology, Children's and Adolescents' Hospital, Cologne, Germany

Kerpener Str. 62, 50937 Köln, Germany

Phone: +49 (0)221 – 478 42101

Fax: +49 (0)221 – 478 5835

Email: rasmus.ehren@uk-koeln.de

Supplementary table 1

patient	sex	age at onset of disease (years)	age at investigation (years)	etiology of aHUS	genetic findings	factor H auto-antibodies	duration of eculizumab therapy before switch to ravulizumab (months)	complications during ravulizumab therapy	additional therapy
**1**	female	7	8	DEAP-HUS	CFHR1/2del, homozygous	positive	6	none	mycophenolate mofetil
**2**	male	0.7	18	CFH mutation	c.2770T>A, homozygous	negative	123	none	none
**3**	male	0.5	10	CFH mutation	c.2827_2831delGCTCA, heterozygous	negative	103	none	none
**4**	female	0.4	9	CFH mutation	c.3565C>T, heterozygous	negative	74	none	none
**5**	male	15	20	C3 mutation	c.481C>T, heterozygous	negative	53	none	none
**6**	female	0.4	6	CFH mutation	c.3644G>A, heterozygous	negative	65	none	none

Supplementary table 1: Characteristics of the six patients with genetically and clinically proven atypical hemolytic uremic syndrome. (aHUS, atypical hemolytic uremic syndrome; C3, complement factor 3; CFH, complement factor H; CFHR1/2, complement factor H-related proteins 1/2; DEAP-HUS, deficiency of CFHR plasma proteins and autoantibody-positive form of hemolytic uremic syndrome; del, deletion)
