# Supplementary material for: Real-world data of six patients with atypical hemolytic uremic syndrome switched to ravulizumab
Source: Pediatr Nephrol. 2021 Jul 17;36(10):3281–2. doi: 10.1007/s00467-021-05203-8 (PMC8445866; doi:10.1007/s00467-021-05203-8)
Supplement: Supplementary file 2 — (PDF 561 kb) [file 467_2021_5203_MOESM2_ESM.pdf]

Letter to the Editors

# Real-world data of six patients with atypical hemolytic uremic syndrome switched to ravulizumab

Rasmus Ehren[1], Sandra Habbig[1]

[1] University of Cologne, Faculty of Medicine and University Hospital Cologne, Department of Pediatrics, Cologne, Germany

Corresponding author

Dr. Rasmus Ehren

Pediatric Nephrology, Children's and Adolescents' Hospital, Cologne, Germany

Kerpener Str. 62, 50937 Köln, Germany

Phone: +49 (0)221 – 478 42101

Fax: +49 (0)221 – 478 5835

Email: rasmus.ehren@uk-koeln.de

<u>Supplementary figure 1</u>

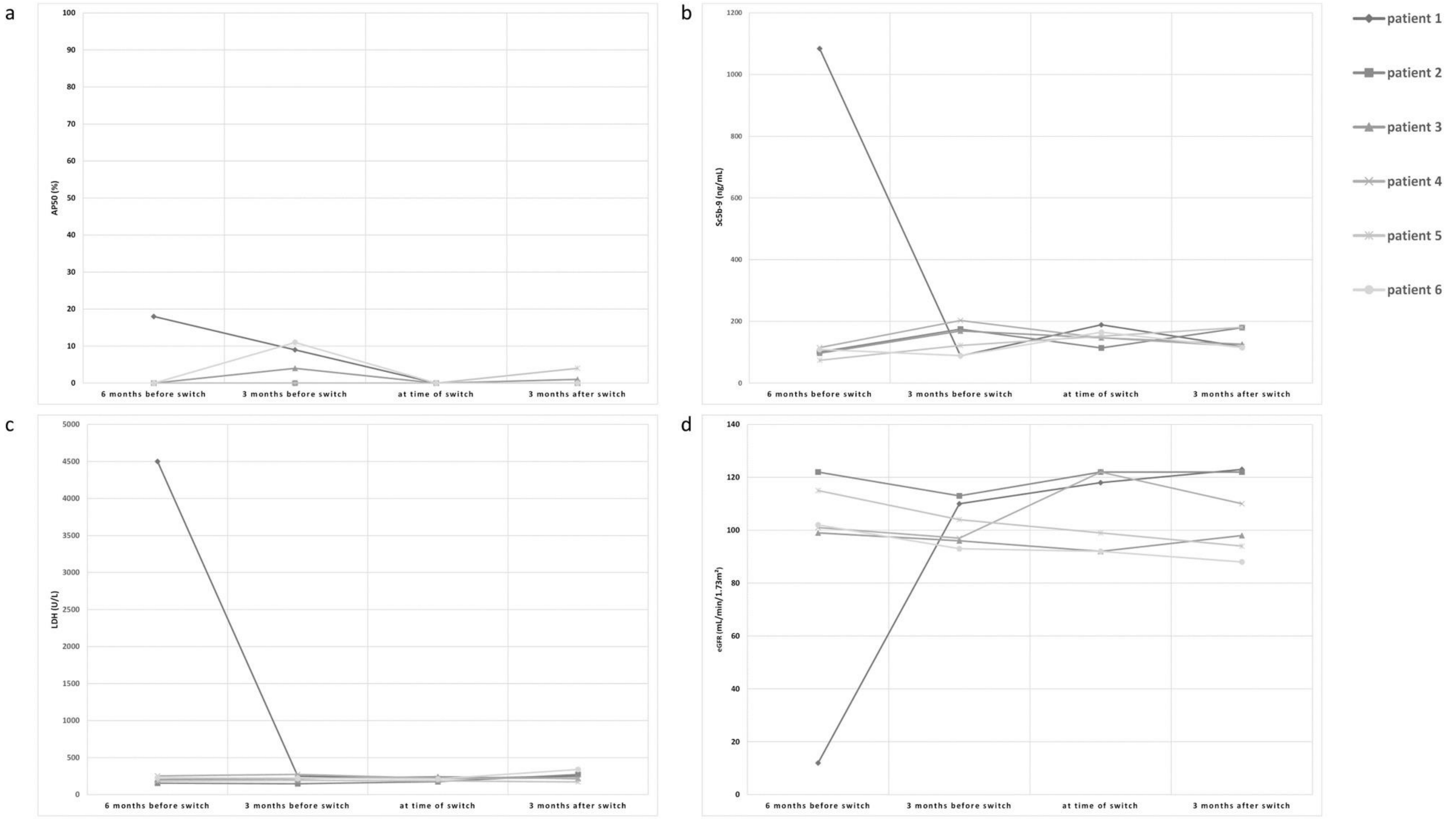


**Supplementary figure 1:** Data of six patients on a) AP50 activity, b) sC5b-9 levels, c) LDH values and d) eGFR according to the full age spectrum (FAS) formula before and after the switch from eculizumab to ravulizumab. Note: Patient 1 developed aHUS just before the "6 months before switch" time point. (AP50, alternative complement pathway; eGFR, estimated glomerular filtration rate; LDH, lactate dehydrogenase; sC5b-9, soluble terminal complement complex)
